# Listeriosis in Portugal: an existing but under reported infection

**DOI:** 10.1186/1471-2334-6-153

**Published:** 2006-10-20

**Authors:** Gonçalo N Almeida, Paul A Gibbs, Tim A Hogg, Paula C Teixeira

**Affiliations:** 1 Escola Superior de Biotecnologia, Universidade Católica Portuguesa, Rua Dr. António Bernardino de Almeida, 4200-072 Porto, Portugal

## Abstract

**Background:**

Listeriosis is a rare disease caused by the bacterium *Listeria monocytogenes*, the normal vehicle of which is food. The disease, which is largely confined to its risk groups of pregnant women, the elderly and immunocompromised individuals, has increased in incidence in recent years.

In Portugal, listeriosis is not a notifiable infection and available data are scarce.

The objective of this work was to collate the available information concerning listeriosis in Portugal by compiling a retrospective study of cases recorded over a decade.

**Methods:**

Requests for case data on clinically confirmed listeriosis, recorded over the previous decade, were replied to by 23 hospitals and a National Institute of Health delegation.

**Results:**

35 cases of listeriosis were identified for the period between 1994 and 2003 inclusive, the mortality rate being greater than 17%. According to the data collected in this study for the year 2003, the incidence of this disease in Portugal was at least 1.4 cases per million inhabitants in that year.

**Conclusion:**

The study demonstrates, for the first time in the widely available literature, that despite their being no cases of listeriosis in Portugal recorded in official reports, the threat of *L. monocytogenes *to public health is of a similar dimension to that in other countries.

## Background

*Listeria monocytogenes *is an ubiquitous bacterium responsible for cases and outbreaks of listeriosis in humans and animals, normally transmitted by consumption of contaminated foods or feeds [1]. In recent years several outbreaks of listeriosis have been described, associated with the consumption of a wide variety of foods, ranging from dairy products, to ready-to-eat deli meats [1].

Although exposure to the bacterium is common [2], listeriosis is rare within the general population; incidence in Europe varies between 0.3 and 7.5 cases per million inhabitants with a mortality rate between 20 and 30% [3]. Certain groups within the general population are particularly susceptible to infection, namely immunocompromised persons, (e.g. organ transplant or cancer patients), HIV-infected individuals, pregnant women, newborn babies and the elderly [1]. In Portugal, listeriosis is not a notifiable infection and available data are scarce [4]. In a recent published document [3], it is stated "... all participating countries except Portugal have at least one surveillance system for listeriosis". Moreover, in a publication of the Portuguese National Health Services concerning advice for the prevention of infectious diseases during pregnancy [5], *L. monocytogenes *is not mentioned.

The present study aimed to contribute to the knowledge of listeriosis in Portugal through compiling a retrospective study of the cases identified over the period between 1994 and 2003, inclusive.

## Methods

Data were obtained from requests to the Clinical Pathology Services of 23 hospitals and the National Institute of Health's delegation in Porto. Correspondence was received during the period between October 2003 and February 2004. Retrospective identification of cases of listeriosis during the previous decade was requested from each hospital. The contact person in each hospital was requested to complete a form containing the following information regarding each case: year of isolation, age and sex of the patient, factors that increased risk, the tissue or fluid origin of the isolate and the outcome of the infection. For the purposes of this study a case of listeriosis is defined as when clinical symptoms were consistent with a systemic infection with this organism and *L. monocytogenes *is isolated from a normally sterile site (usually blood or cerebrospinal fluid, or less commonly, joint, pleural, or pericardial fluid) or from placental or foetal tissue in the setting of miscarriage or stillbirth and. Approval by an Ethics Committee was not required.

Results

At least 35 cases were identified between 1994 and 2003 (Table 1). The highest annual number of cases was recorded for the year of 2003, this might be explained by the fact that the hospitals were contacted in October of that year and were obviously more vigilant in the detection and recording of this infection.

**Table 1:** Listeriosis in Portugal: 1994–2003

**Date of isolation**	Age†/Sex*†	**Predisposing factors†**	**Clinical manifestations†**	**Isolated from†‡**	**Clinical outcome†**
November 1994	New-born/M	Age	DNR	Liver	Fatal
1996	DNR/M	HIV	Fever	Blood and CSF	DNR
July 1997	DNR	DNR	DNR	Blood	DNR
September 1997	DNR	DNR	DNR	CSF	DNR
October 1997	DNR	DNR	DNR	CSF	DNR
1998	54/M	DNR	DNR	Blood	DNR
April 1998	DNR	DNR	DNR	CSF	DNR
January 1999	DNR	DNR	DNR	CSF	DNR
July 1999	DNR	DNR	DNR	CSF	DNR
September 1999	DNR	DNR	DNR	Blood	DNR
October 1999	New-born/F	Age	DNR	Lung	Fatal
2000	48/M	DNR	Fever and headache Stiff neck	CSF	DNR
2000	25/F	Pregnancy	Flu syndrome 15 days before birth	Vaginal culture	Birth at 36 weeks of pregnancy
2000	New-born/DNR	Age	Hypotonia, breath difficulties, sepsis at birth. Apgar score 5 (1 min) – 7 (5 min)	Blood	Favourable without sequelae
March 2000	DNR	DNR	DNR	Blood	DNR
April 2000	DNR	DNR	DNR	Blood	DNR
June 2000	DNR	DNR	DNR	CSF	DNR
October 2000	DNR	DNR	DNR	Blood	DNR
May 2001	41/M	Cirrhosis	DNR	Blood	Fatal
October 2001	85/M	Age	DNR	Blood	Fatal
February 2002	DNR/M	DNR	Meningitis	CSF	DNR
March 2002	55/M	Haematological illness.	DNR	Blood	Favourable
2003	75/F	Chronic renal failure	DNR	Blood	Favourable
2003	New-born/DNR	Age	DNR	Blood	Favourable
2003	DNR/F	DNR	DNR	Vaginal culture	DNR
2003	DNR/F	DNR	DNR	Vaginal culture	DNR
January 2003	69/M	Age	DNR	Blood	Favourable
February 2003	74/F	Age	Meningitis	CSF	Favourable
February 2003	31/F	Pregnancy	DNR	Placenta	Favourable
April 2003	New-born/DNR	Age	DNR	Blood	Favourable without sequelae
April 2003	67/M	Corticosteroid therapy. Nephritic syndrome	Meningitis	Blood, CSF and ascitic fluid	Fatal (Septic shock)
May 2003	25/M	DNR	Meningitis	CSF	DNR
July 2003	85/F	Age	DNR	CSF	DNR
October 2003	50/M	Alcoholism	DNR	Blood/CSF	Fatal
November 2003	48/M	DNR	DNR	CSF	With internment return but without sequelae

†DNR, Data not recorded.

*F, Female; M, Male.

‡CSF, cerebrospinal fluid.

The age of the patients, reported for 20 out of the 35 cases, varied between neonates (5 new-borns, 25%) and 85 years (6 older than 65 years, 30%), and were predominantly male (13 out of the 21 cases recorded, 62%). For 7 cases other malignancies were recorded and included; cirrhosis, haematological syndromes, alcoholism, chronic renal failure, nephritic syndrome and HIV infection (Table 1).

In the present study, even though the clinical symptoms were not recorded for the majority of cases, isolation of *L. monocytogenes *from blood (40%) and from cerebrospinal fluid (43%) was positive. The clinical signs described were meningitis, fever and sepsis at birth. The clinical outcome, recorded in 16 episodes, was fatal for 6 patients. Therefore the observed death rate must lie between 17% (6/35 – the total number of listeriosis episodes reported) and 37,5% (6/16 – the number of listeriosis episodes for which full case histories are available). Patients with a favourable clinical outcome apparently did not present any sequelae.

Discussion

According to the data collected in this study, the incidence of listeriosis was at least 1.4 cases per million inhabitants for the year 2003 considering the resident population of Portugal as that given by the National Institute of Statistics. This relatively low incidence must be considered with care because it is taken from a single year and the data gathered certainly cannot be considered exhaustive. Notification of listeriosis is not legally obligatory in Portugal. This, along with the fact that clinical symptoms are sometimes not evident, thus making diagnosis difficult, is an impediment to the gathering of more credible data [6-8]. Considering that not all of the country's healthcare units were contacted in this study, the national incidence rate presented here is, of necessity, an initial estimate.

From the recorded case data, *L. monocytogenes *was mainly isolated from blood and cerebrospinal fluid. It has been reported in a study with similar scope that, in cases where non-perinatal infection has led to clinical manifestations, primary bacteraemia was the most common cause (47%) followed by meningitis (28%) [7].

As reported for other countries, the majority of the cases were non-perinatal [7]. The case-fatality rate, similar to that reported in studies from other countries, 36% worldwide average for non-perinatal cases [7] and 40% for Spain [9], reflects the severity of the infection, in particular among new-borns and immunocompromised. As in previous studies, a predominance of the infection in males was verified [7,8].

## Conclusion

The study demonstrates, for the first time in the widely available literature, that despite their being no cases of listeriosis in Portugal recorded in official reports, the threat of *L. monocytogenes *to public health is of a similar dimension to that in other countries [3,4].

It is highly likely that there will be a continued increase in the size of certain at-risk groups, namely the elderly and immunocompromised patients. The education of these and other, at-risk groups and of the professionals providing care for them, is proposed as a key strategy in the reduction of the incidence of listeriosis [10]. An example of this need is the fact that in a survey of 312 women that had been pregnant, only 54% changed their food habits during pregnancy (P. Teixeira, unpublished data).

## Competing interests

The author(s) declare that they have no competing interests.

## Authors' contributions

GNA and PCT conceived the study, data collection and analysis. PCT drafted the manuscript. PAG and TAH revised the manuscript critically.

All authors read and approved the final manuscript.

## Pre-publication history

The pre-publication history for this paper can be accessed here:


